# The Influence of Alloy Composition on Microstructure and Performance of Mixed-Smelting Alloy and Weld Metal

**DOI:** 10.3390/ma17194708

**Published:** 2024-09-25

**Authors:** Guangnan Ge, Jin Hu, Zongqiu Hu, Haijun Li, Yan Huo, Shawei Tang, Yi Liu, Junfeng Ding, Shipu Hou, Yunbao Gao

**Affiliations:** 1 Department of Materials Science and Engineering, Harbin Institute of Technology, Harbin 150001, China; clggn_0603@163.com; 2 State Key Laboratory of Hydro-Power Equipment, Harbin 150040, China; huoy@hec-china.com (Y.H.); liuyi@hec-china.com (Y.L.); dingjf@hec-china.com (J.D.); hsp@hec-china.com (S.H.); 3 Harbin Institute of Large Electric Machinery Company Limited, Harbin 150040, China; 4 China Three Gorges Construction Engineering Corporation, Beijing 101100, China; hu_zongqiu@ctg.com.cn (Z.H.); li_haijun@ctg.com.cn (H.L.); 5 National Key Laboratory of Advanced Casting Technologies, Shenyang 110022, China; gyb1394005869@163.com

**Keywords:** mixed smelting, microstructure, corrosion resistance, heat input, fusion ratio

## Abstract

In the present work, the Q345B low-alloy steel with different contents and ER309L stainless steel were melted together to obtain new alloys. The aim was to design the composition of weld metal (Q345B low-alloy steel as the base material and ER309L welding wire as the filler material) and improve the corrosion resistance of the weld metal. During the welding process, the composition of the weld metal was controlled to match the new alloys by changing the welding heat input. A relationship curve between fusion ration and welding heat input was obtained. The research focused on analyzing the effect of mixed-smelting ratio between Q345B and ER309L and welding heat input on the microscopic structure and corrosion performance of the prepared samples. The results show that the melted alloys containing 20% to 30% Q345B consist of a ferrite (δ) phase and austenite (A) phase, the samples containing 45% to 50% Q345B consist of a martensite (M) phase and austenite (A) phase, and the sample containing 40% Q345B consists of a martensite (M) phase, ferrite (δ) phase, and austenite (A) phase. As the mixed-smelting ratio of Q345B/ER309L increased, the corrosion resistance of samples decreased gradually. For the weld metal, the fusion ration between Q345B base material and ER309L welding wire increases with the welding heat input. When the heat input changed from 0.645 kJ/mm to 2.860 kJ/mm, the composition of the weld metal was consistent with the melted alloys containing 20–45% Q345B. The microstructure and corrosion resistance of the weld metal could be designed by the melting means, which has important guiding significance for engineering applications.

## 1. Introduction

Steel has become one of the most commonly used materials, due to its low price and reliable performance, and is widely used in the construction, manufacturing, and medical equipment fields [1,2]. Austenitic stainless steel has excellent mechanical properties and good corrosion resistance and weldability, and is widely used in marine environmental equipment and the petrochemical fields [3,4,5,6,7,8]. Low-alloy steel has been commonly applied in the manufacturing of nuclear power and marine components, due to its good characteristics, such as plasticity, toughness, and welding performance [9,10,11]. Due to the advantages of combining both materials and the reduction in manufacturing costs, the dissimilar welded joint of the above two types of steels is widely used in the manufacturing of power-generation equipment and oil pipelines [12,13,14]. The Q345B low-alloy steel has a low price and good performance advantages. It is commonly used in structural engineering and manufacturing [15]. ER309L welding wire has good heat and corrosion resistance, thermal stretchability and welding arc stability [16,17]. It is widely used in the welding of stainless steel and low-alloy steel, as well as repairing defects in low-alloy steel [18]. Therefore, ER309L welding wire is often used as a filler material for Q345B low-alloy steel and austenitic stainless steel.

In order to further reduce manufacturing costs and meet the requirements of a corrosion-performance environment, there are many articles reporting studies about the corrosion resistance of dissimilar welded joints of low-carbon steel and stainless steel. Abioye et al. [19] used gas-shielded metal arc welding to study the microscopic structure and corrosion performance of dissimilar welded joints of low-carbon steel and AISI 304 stainless steel under different welding speeds. They found that the corrosion performance improved as the welding speed decreased. Huang et al. [20] explored the influence of arc energy on the microstructure and corrosion performance of weld metal between low-carbon steel and a stainless steel welded joint. It was found that higher arc energies could obtain the best corrosion resistance of the weld metal. Rahman et al. [21] used the heat inputs of 1 kJ/mm, 3 kJ/mm, and 5 kJ/mm to study the corrosion resistance of dissimilar steel weld metals. They found that the rate of pitting and crevice corrosion in the weld metal with heat inputs of 1 kJ/mm and 3 kJ/mm were higher, while the rate in the weld metal with 5 kJ/mm was lower. There was no doubt that the welding process can significantly affect the microstructure of weld metal and then affect the performance of welded joints.

In practical applications, due to the different compositions and structures of weld metal and base material, the welded joints are prone to failure in service [22,23,24,25,26]. In order to save costs, repair welding is generally carried out on defected welded joints in engineering. Many scholars have researched the influences of repair welding and multi-layer multi-pass welding on the microscopic structure and corrosion resistance of stainless steel and low-alloy steel dissimilar welded joints [27,28,29,30,31,32,33]. During the multi-layer multi-pass welding process of dissimilar steel, the phase composition of the weld metal is extremely complex, due to the multiple melting and solidification of the weld metal. The corresponding corrosion resistance mechanism is difficult to unify. In order to eliminate the influence of multiple thermal cycles during the multi-layer multi-pass welding process, we have adopted the method of single-layer single-pass welding to investigate the microstructure and corrosion performance of the weld metal under different heat inputs [34]. We found that the arc energy had a visible effect on the content of ferrite and austenite phases, which affected the corrosion resistance of the weld metal. Due to the chemical composition of the base material and welding material being similar in our study, the composition of base material and weld metal did not change significantly.

In nature, to fully utilize the performance advantages of different materials and reduce the manufacturing costs, dissimilar steel-welded joints play an important role in the industrial field [29,30,35,36]. The welded joint of low-alloy steel and stainless steel has been applied to a certain component of tidal units. The complex marine environment and the operational characteristics of the tidal units propose higher demands for the performance of the welded joint. However, when two different types of steels are welded together, the phase composition of weld metal is reasonably complex. This makes it difficult to determine the constitutive relationship between microstructure and performance of the weld metal. It invisibly affects the safety and service life of the welded joint. It is necessary to design the components of weld metal before welding, in order to avoid a lot of actual welding work.

In the present work, the new alloy composition was the design. The Q345B low-alloy steel and ER309L austenitic stainless steel were melted in a vacuum arc melting furnace. During the melting, the contents of Q345B were different, in order to obtain the new alloys with different composition proportions. Different proportions of fusion were performed on the Q345B and ER309L to reflect the changes in the weld metal of the base metal caused by different heat inputs. It was hoped that each new alloy prepared would represent the weld metal with a fusion ratio. The influences of alloy composition on microscopic structure and corrosion performance of mixed-smelting Q345B and ER309L were systematically studied. It could provide an experimental basis and theoretical support for the multi-layer multi-pass welding and repair welding of dissimilar steel-welded joints. The ER309L welding wire was deposited on the Q345B plate using the GMAW method and different welding heat inputs. This article verifies the microstructure and corrosion resistance of weld metal with the same composition as the designed alloy. This article provides a theoretical basis for designing and improving the corrosion resistance of the low-alloy steel and stainless steel dissimilar-welded joints, thereby enhancing their service life in engineering applications. In particular, a relationship curve between fusion ratio and welding heat input has been established. Low-alloy steel and austenitic stainless steel dissimilar-welded joints that met usage requirements could be prepared.

## 2. Experimental Section

### 2.1. Materials

The base material was a Q345B low-alloy hot-rolled steel sheet with dimensions of 16 mm × 100 mm × 200 mm. The filler material was AWS A5.9 ER309L welding wire with a diameter of 1.2 mm. The chemical composition of the base metal and welding wire are shown in Table 1.

### 2.2. Mixed-Smelting and Welding Conditions of Q345B and ER309L

**Figure 1:**
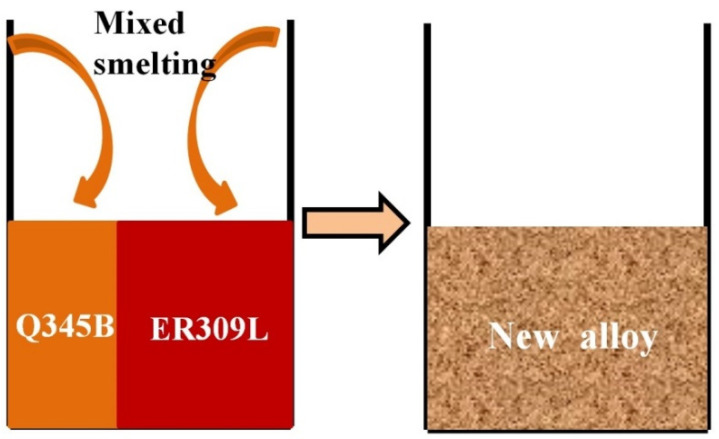
Schematic diagram of Q345B and ER309L mixed melting.

The Q345B low-alloy and AWS A5.9 ER309L were melted using a KDH-500 non-consumable vacuum arc melting furnace, as shown in Figure 1. The contents of Q345B were 20%, 30%, 40%, 45%, and 50%, respectively.

**Figure 2:**
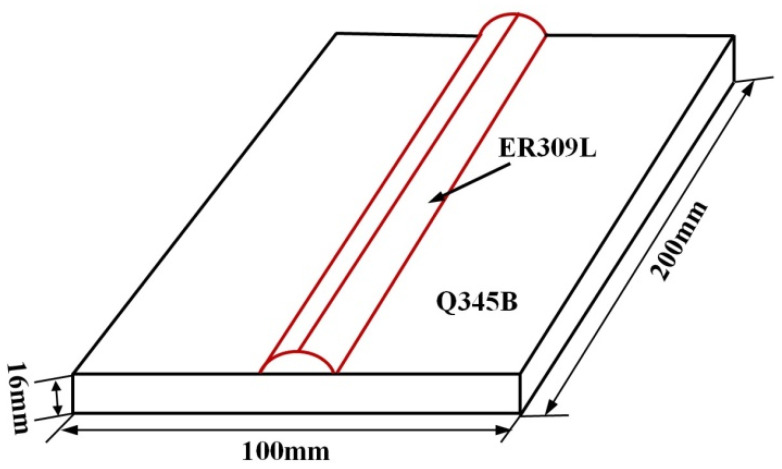
Schematic diagram of the welding process.

AWS ER309L welding wire was deposited on Q345B low-alloy steel plate, and the deposit method was gas metal arc welding (GMAW) (see Figure 2). The protective gas used during the welding process was 95% argon and 5% carbon dioxide, and the flow rate of the gas was 20 L/min. The calculation formula for welding heat input was as follows [37]:

(1) $$H=\eta \;\left(U\times Ι\right)/\nu$$

where *H* is arc energy, measured in J/mm; U is voltage, measured in V; *I* is current, measured in A; ν is welding speed, measured in mm/s; and η is welding efficiency. The η of GMAW is 0.8. A low heat input can easily cause incomplete fusion and defects in the welded joint, due to the insufficient energy, while high heat input can easily lead to harmful phases in the weld metal, due to the excessive energy. The parameters under different welding processes are shown in Table 2. The heat inputs under different processes are 0.645 kJ/mm, 0.962 kJ/mm, 1.765 kJ/mm and 2.860 kJ/mm, respectively.

### 2.3. Microstructure Examination and Chemical Composition Analysis

The microstructure and chemical composition of melted alloys and weld-metal samples were examined by using an S-3700N scanning electron microscope (SEM) with an energy-dispersive X-ray (EDX) spectroscope made by Mitsubishi Corporation of Japan and ZEISS AXIO optical microscope (OM) made by Deiss Company of Germany. The surface of the studied samples was prepared by grinding, polishing, and etching. Finally, they were etched using aqua regia (ratio of HCl and HNO_3_ is 3:1), to observe the microstructure.

### 2.4. Corrosion Test

The electrochemical testing was conducted using a ZENNUIM computer-controlled potentiostat made by Zanna Company of Germany. All samples needed to be polished, cleaned with alcohol, and then dried in the air. The test areas of the melted samples and the weld metals were 0.785 cm^2^ and 0.3848 cm^2^, respectively. The test temperature was 25 ± 5 °C, and the test medium was 3.5 wt% NaCl solution prepared with distilled water.

The experiments were carried out in a conventional three-electrode system, and the test sample and the saturated calomel electrode (SCE) were the working electrode and the reference electrode, respectively. The graphite rod was as the auxiliary electrode.

Electrochemical impedance spectroscopy (EIS) was tested after the open-circuit potential stabilized for 1800 s. The testing frequency ranged from 100 kHz to 10 mHz. The amplitude of the sine voltage signal during EIS testing was 10 mV.

The Potentiometric Polarization Curve (PC) test was conducted after the open-circuit potential stabilized for 1800 s. The scan voltage started at −0.2 V below the sample’s open-circuit potential, and ended at the current density of 1 mA/cm^2^. The ZSimpWin 3.30 version software was used to fit and analyze the EIS test result.

To ensure the accuracy of the data, all electrochemical tests were carried out more than three times.

## 3. Results and Discussion

### 3.1. Microstructure Characterization of Mixed-Smelting Sample

#### 3.1.1. OM Examination

**Figure 3:**
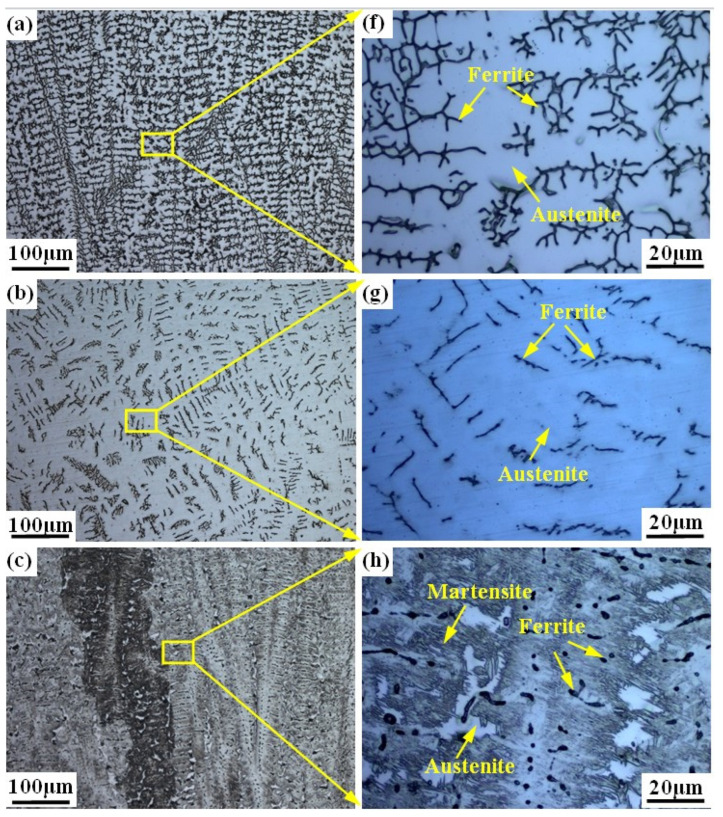

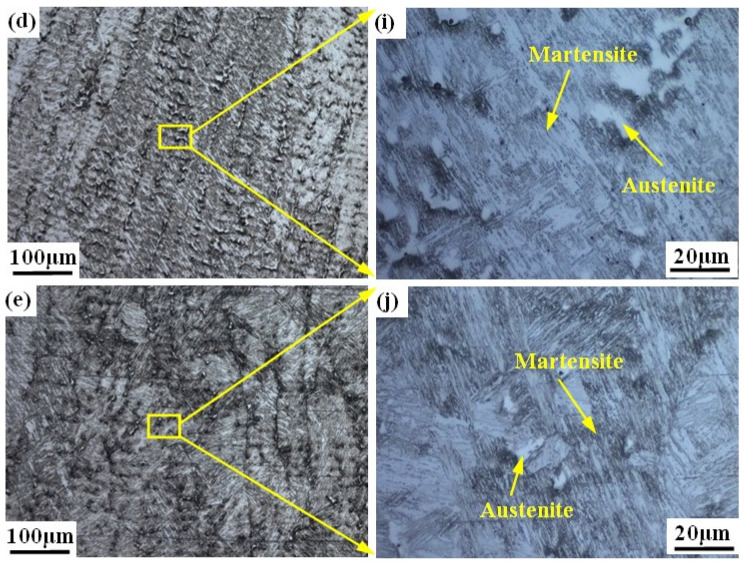
Microstructure of Q345B and ER309L under different smelting ratios: (**a**,**f**) 20% (**b**,**g**) 30% (**c**,**h**) 40% (**d**,**i**) 45% (**e**,**j**) 50%.

Figure 3 shows the microstructure of the prepared alloy samples under different smelting ratios. The columnar crystals can be seen in all samples (see Figure 3a–e). The microstructure of samples with smelting ratio of 20% and 30% Q345B/ER309L consists of the ferrite (δ) phase and austenite (A) phase. The δ phase was distributed randomly in the austenite (A) phase matrix (see Figure 3f,g). The microstructure of the sample with the smelting ratio of 40% Q345B/ER309L consists of the martensite (M) phase, ferrite (δ) phase, and austenite (A) phase. The δ phase was distributed in the M phase matrix and the interface between the M phase and A phase (see Figure 3h). The microstructure of samples with 45% and 50% smelting ratios consists of M phase and A phase (see Figure 3i,j). When the sample smelting ratios are 20% and 30%, the morphologies of the δ phases are shown to be dendritic-like and worm-like, respectively (see Figure 3a,b). The δ phase in the sample with the smelting ratio of 40% is distributed at the boundaries between the M phase and A phase, and in the M phase. The contents of the chromium (Cr) element and nickel (Ni) element in ER309L steel is higher than that in Q345B low-alloy steel, but the carbon (C) element content is lower. As the smelting ratio of Q345B/ER309L in the new alloy increases, the content of the Cr and Ni elements gradually decreases, while the content of the C element gradually increases. The change in element content leads to significant differences in the microstructure obtained from the samples under different smelting ratios.

**Figure 4:**
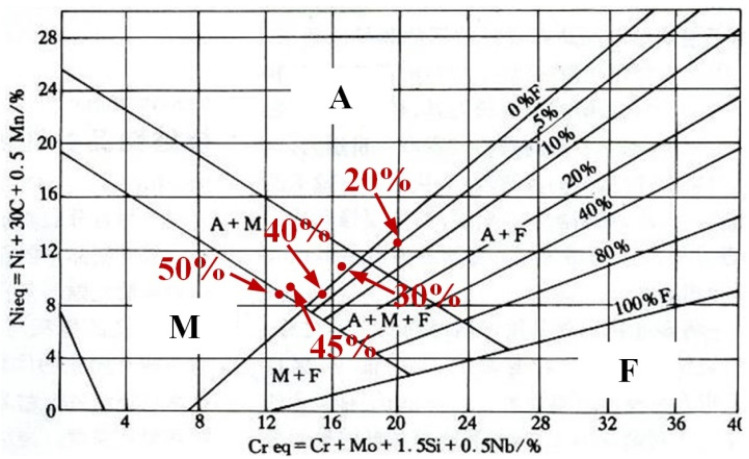
Schaeffler diagram for predicting the microstructure of different samples [36,37].

As shown in Figure 4, the phase composition of the prepared sample can be predicted and analyzed using the Schaeffler diagram [38,39]. In Figure 4, the *x*-axis represents chromium equivalent (Creq) and the *y*-axis represents nickel equivalent (Nieq). The Creq and Nieq of prepared samples are calculated according to the formulas in Figure 4. The calculation results of the prepared samples are displayed in Table 3. As the contents of Q345B increased, the Creq and Nieq in the prepared samples gradually decreased. When these values are input into the Schaeffler diagram, the microstructures of the prepared samples contain different proportions of Q345B. The microstructure of the samples containing 20% and 30% Q345B is very close to the A + F phase region, and the samples containing 45% and 50% Q345B is in the M + F phase region. However, the sample containing 40% Q345B is in the M + A + F phase region. The predicted results for the different samples, according to Figure 4, are consistent with the observations in Figure 3.

#### 3.1.2. SEM Examination and EDS Analysis

**Figure 5:**
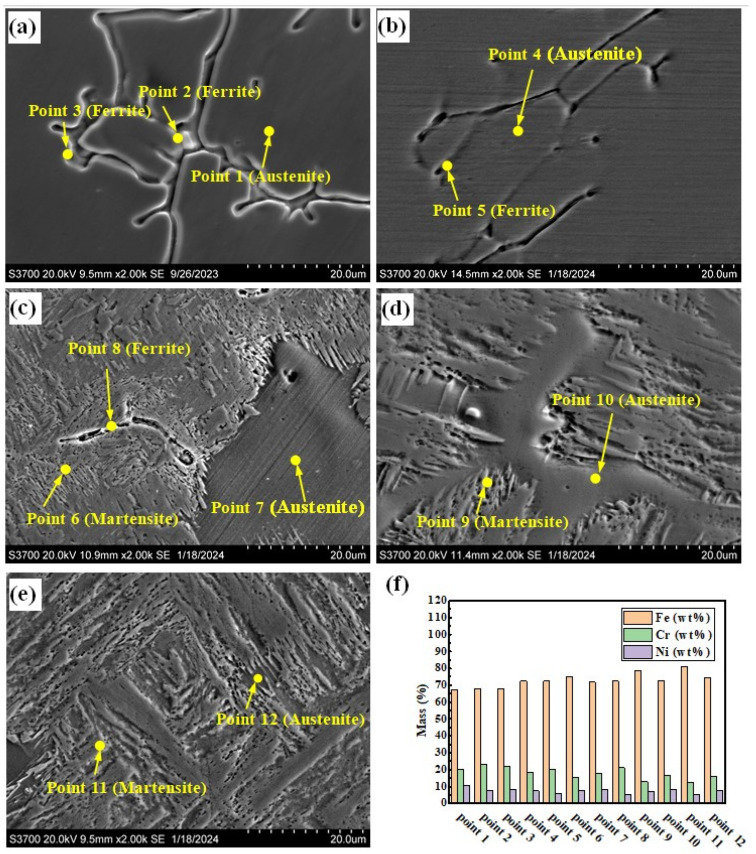
SEM morphologies of the prepared samples under different Q345B contents: (**a**) 20% (**b**) 30% (**c**) 40% (**d**) 45% (**e**) 50%. (**f**) Histograms of elements obtained from the positions shown in (**a**–**e**).

Figure 5a–e show the SEM photographs of the prepared samples. There are two forms of δ phase in the sample with the smelting ratio of 20%: they are dendritic and rod-shaped, as shown in Figure 5a. The A phases in the samples with smelting ratios of 45% and 50% are distributed along the grain boundaries (see Figure 5c,d). Figure 5f shows the histograms of element content obtained from the positions shown in Figure 5a–e. For the samples under different smelting ratios, the Cr element content in the δ phase is higher than that in the A phase and M phase. However, the Ni element content is lower than that of the A phase, as shown in Figure 5f. As the contents of Q345B in the prepared samples increases, the Cr element content in the δ phase remains basically unchanged, while the Ni element content gradually decreases. The Cr element and Ni element contents in the M phase gradually decrease. At the same time, the Cr element content in the A phase gradually decreases, while the Ni element content remains basically unchanged. This may be due to the increase in Q345B content, which leads to the decrease in Cr and Ni elements in the sample. It changes the formation conditions of the δ phase, M phase, and A phase.

### 3.2. Corrosion Properties for Mixed-Smelting Sample

**Figure 6:**
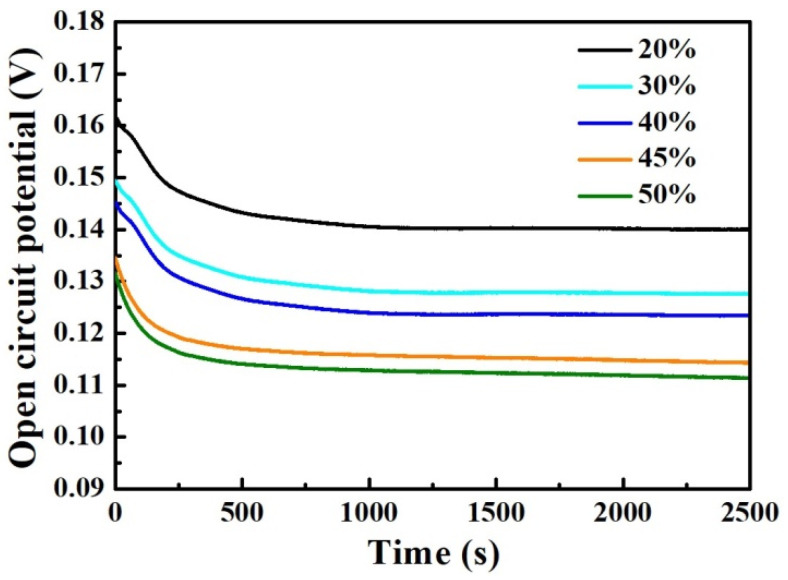
Open-circuit potential vs. time curves for the prepared samples under different Q345B contents immersed in the 3.5 wt% NaCl solution.

Figure 6 shows the variation curves of open-circuit potential over time for the mixed smelting samples in 3.5 wt% NaCl medium. As is well known, the open-circuit potential can reflect the corrosion characteristics of the samples. It can be seen that the OCP-t curves for the different samples exhibit the same characteristics. During the soaking process, the OCP curves of all samples gradually decrease at the beginning of soaking, and then tend to stabilize. As the content of Q345B increases in the melted samples, the OCP values of all samples gradually decrease, which means that the corrosion resistance of the sample decreases.

**Figure 7:**
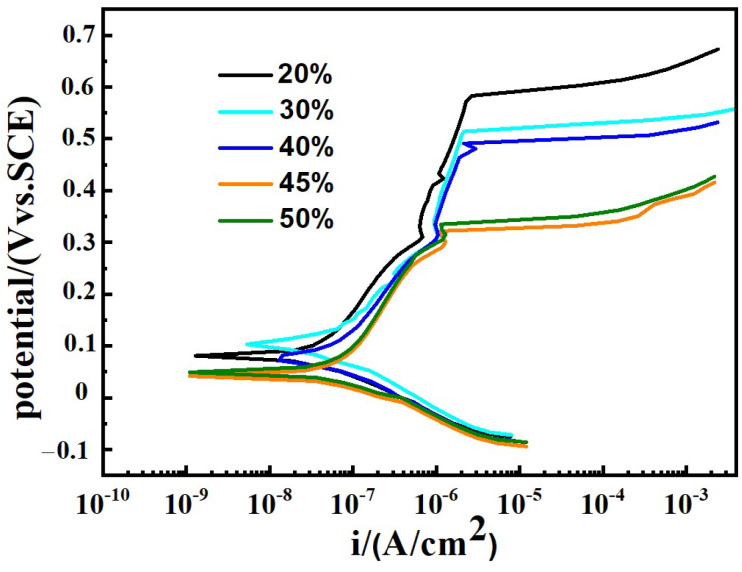
Potentiodynamic polarization curves of prepared samples under different Q345B contents in the 3.5 wt% NaCl solution.

Figure 7 presents potentiodynamic polarization curves of samples under different Q345B contents in the 3.5 wt% NaCl solution. From Figure 7, it can be seen that the polarization curves of all samples exhibit the same characteristics. There is an obvious passivation zone on the anodic polarization curves of all samples, indicating that the passivation film has been formed on the surface of all samples in the air [40].

Table 4 shows the data fitted from Figure 7. As the content of Q345B increases, the corrosion potential (*E*_corr_) of prepared samples gradually decreases, while the corrosion current density (*I*_corr_) gradually increases. The breakdown potential (*E*_b_) can be used to evaluate the local corrosion behavior of metal alloy. As is well known, the parameter *E*_b_–*E*_corr_ reflects the width of the passivation zone on the polarization curve and the sensitivity to pitting corrosion resistance. From Table 4, it can be found that the extent of the passive region gradually decreases with the increase in Q345B content. It means that the pitting corrosion resistance gradually decreases with the increase in Q345B content.

The ER309L has a high content of Cr and Ni elements, and there are almost no Cr and Ni elements in the Q345B low-alloy steel. The pitting corrosion resistance of steel is mainly determined by the content of the Cr, Mo and N elements [41,42,43,44]. For the prepared samples, the increase in Q345B leads to the decrease in Cr and Ni contents, while the contents of the Mo and N elements show almost no change. Therefore, the higher the content of Q345B, the lower the content of the Cr element, and the lower the pitting corrosion resistance will be. The sample with 20% Q345B has the highest *E*_b_ and *E*_b_ − *E*_corr_ value, which shows the best performance.

**Figure 8:**
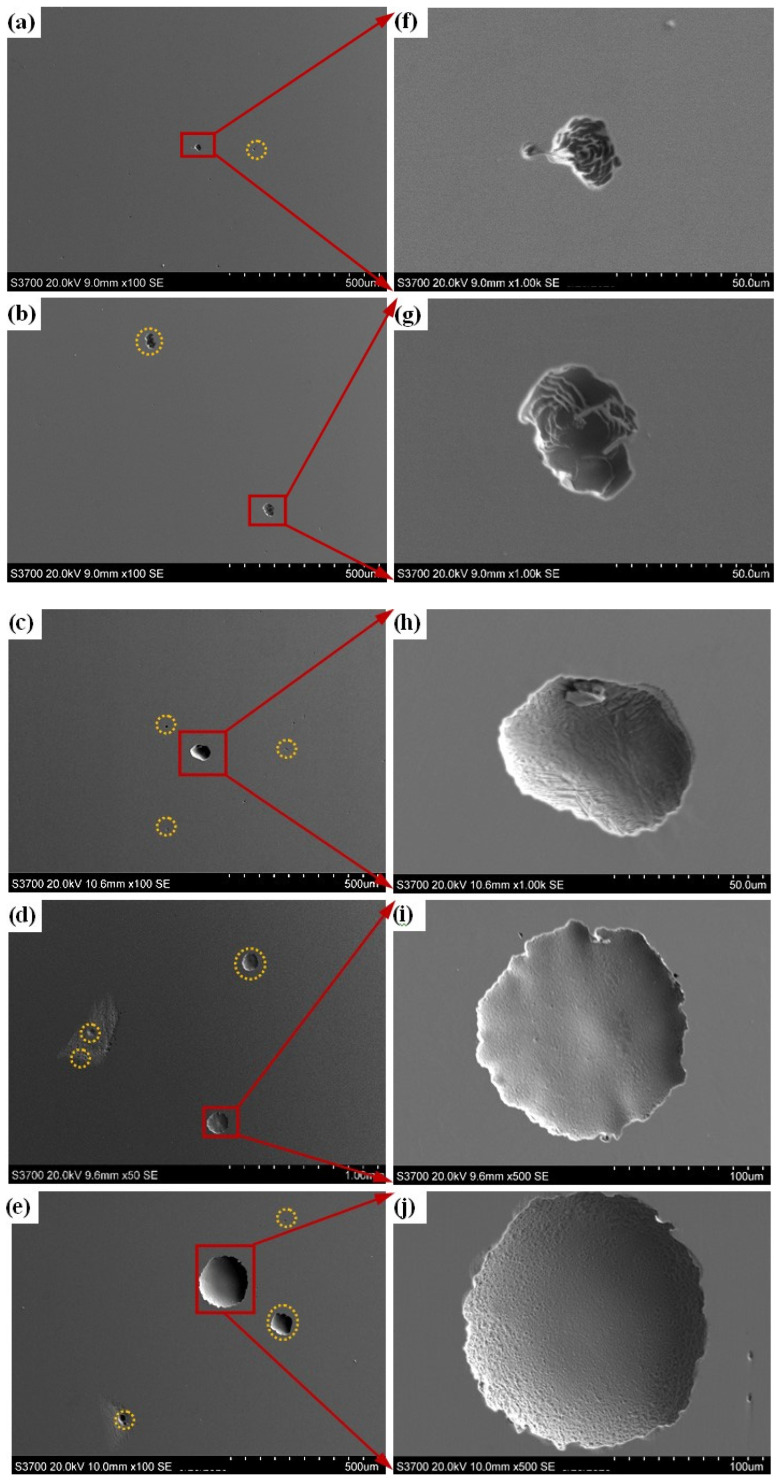
SEM images of the prepared samples under different Q345B contents after polarization: (**a**,**f**) 20% (**b**,**g**) 30% (**c**,**h**) 40% (**d**,**i**) 45% (**e**,**j**) 50%.

Figure 8 shows the SEM images of the prepared samples after the polarization test. Corrosion pits with the different sizes can be observed on the surfaces of all samples. As the content of Q345B in the prepared samples increases, the size and number of corrosion pits gradually increase (Figure 8a–e). As shown in Figure 3, the microstructure of samples containing 20% and 30% Q345B corresponds to the A + δ phases. Due to the formation of the δ phase, the Cr element is lower in the interface between the δ phase and A phase. A pit first forms at the interface between the δ phase and the A phase, and gradually increases around the δ phase and spreads to the A phase [45]. The expansion and connection of multiple small pits form large pits (Figure 8f–j). For the smelted samples with 40% to 50% Q345B contents, the Cr and Ni elements in the melted alloy gradually decrease, resulting in a gradual decrease in their content in the M matrix phase. It means that the pitting corrosion resistance of the matrix is reduced, causing more and larger pits to appear on the surface of the samples (Figure 8h–j).

**Figure 9:**
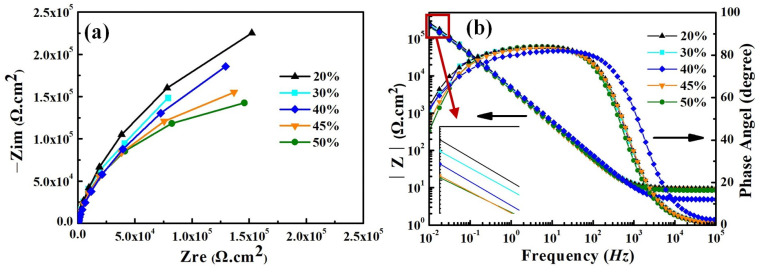
Electrochemical impedance spectra for the prepared samples under different Q345B contents in the 3.5 wt% NaCl solution; (**a**) Nyquist plots, (**b**) Bode plots.

Figure 9 shows the electrochemical impedance spectra (EIS) of the smelted samples in the 3.5 wt% NaCl solution. The EIS of all samples exhibits the same characteristics, and they all own one capacitive loop throughout the entire frequency-testing range. It indicates that they exhibit one time constant during the testing process. No Warburg impedance features are found in the Nyquist plots, and therefore the influence of diffusion impedance on the corrosion behavior of the samples can be ignored. This means that the electrochemical reactions on all sample surfaces dominate the entire corrosion process. The capacitance arc in the impedance diagram can be seen to show that the charge-transfer resistance and double-layer capacitance dominate the entire corrosion process (see Figure 9a). With the increase in Q345B content, the capacitance arc radius of the samples shows the gradually decreasing trend. The Bode plots of all smelted samples have the same characteristics, with only one time constant (see Figure 9b). The slope of log |Z| relative to log f for all samples is approximately −1 in the low-frequency and mid-frequency regions, and 0 in the frequency range of 10^4^–10^5^ Hz. In addition, within the frequency range of 0–10^2^ Hz, the phase angles of all samples are close to 90°, indicating that charge transfer plays a dominant role in the low-frequency region.

As is well known, the size of the capacitance arc radius is related to the corrosion resistance of the melted-alloy samples. As the content of Q345B increases, the capacitance arc radius in the Nyquist plot gradually decreases. This phenomenon indicates that the increase in Q345B content reduces the corrosion resistance of the prepared samples, which is consistent with the results of the potentiodynamic polarization curve.

**Figure 10:**
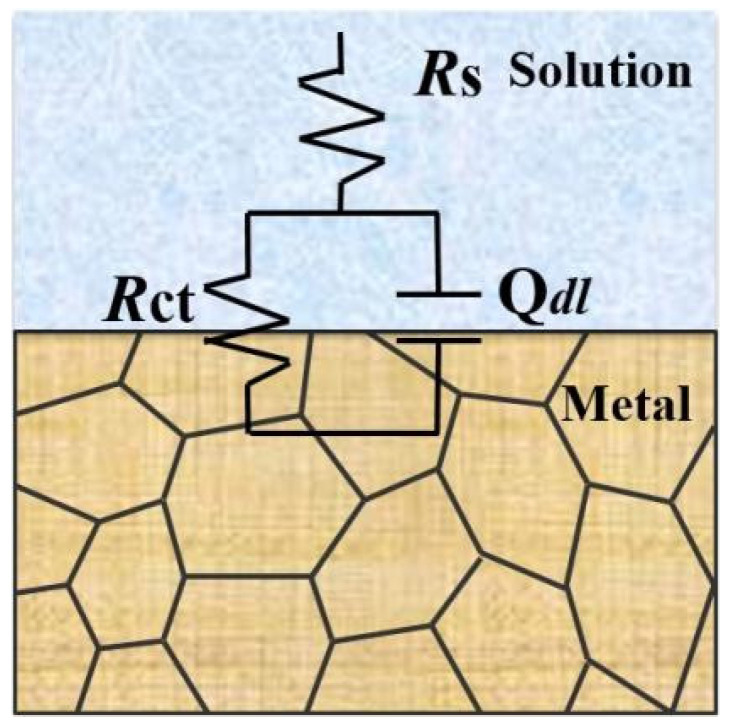
Equivalent circuit fitting of the prepared samples under different Q345B contents in the 3.5 wt% NaCl solution.

The equivalent circuit of EIS is used to reflect corrosion action of metal-alloy materials in various mediums [16]. The equivalent circuit obtained by fitting from Figure 9 is shown in Figure 10. The result obtained from the equivalent circuit is an *R − C* equivalent circuit with one time-constant element, representing the parallel connection of resistor *R*_ct_ and capacitor C. The empirical impedance resistance Z (ω) can be expressed by the following formula [46]:

(2) $$Z\left(\omega \right)=-R_{s}+1/(1/R_{ct}+i\omega C)$$

where ω is the angular frequency, measured in rad/s and ω = 2πf; f is the frequency, measured in Hz; *R*_s_ is the resistance of the solution; *Q*_dl_ is a constant-phase element corresponding to the double-layer capacitor and *Q*_dl_ = *R*^n−1^$C_{dl}^{n};$ and *R*_ct_ is the charge-transfer resistance. In equivalent circuits, the constant phase element (CPE) *Q* is often used instead of the pure capacitor (C) [47].

Table 5 shows the equivalent circuit fitting data results, which describe the corrosion action on the surface of samples. There is good agreement between the measured (symbolic) experimental values and the fitted (solid line) curve (see Figure 9). The range of chi square (χ^2^) values in Table 5 is 8.6 × 10^−5^ − 4.27 × 10^−4^, indicating that the fitted data have a good fitting accuracy. According to Table 5, *R*_ct_ evidently decreases and *Q*_dl_ gradually increases with the increase in content of Q345B. As shown in Figure 9b, a maximum |z| is obtained in the low-frequency range, and the maximum |z| gradually decreases with the increase in content of Q345B. It means that the corrosion resistance of the smelted samples decreases with the increase in the Q345B content. The sample with 20% Q345B content has the greatest charge-transfer resistance, and its corrosion resistance is the best.

### 3.3. Microstructure Characterization of Weld-Metal Sample

During the welding process, the welding wire is melted and deposited between dissimilar steels. Under the high temperature of the welding wire, the base material is melted. Under the action of arc blowing force and oscillation, the welding wire and melted base material are mixed together, forming a relatively uniform molten pool. After the molten pool cools to room temperature, the weld metal is formed. The proportion of melted base metal in the weld metal is called the fusion ratio.

The proportion of base metal fusion is directly related to the amount of melted welding wire. The more melted welding wire there is, the more melted base material there is, resulting in the higher fusion ratio of the weld metal. The welding-process parameters directly affect the amount of melted welding wire. Although there are many welding process parameters, the calculated welding-process parameters correspond to heat input. Therefore, studying the relationship between heat input and fusion ratio is of great significance for the formulation and adjustment of welding-process parameters.

Due to the Q345B studied in this article containing almost no Cr and Ni elements, the fusion ratio can be obtained by the content of the Cr or Ni elements in the weld metal. The fusion ratio under different heat inputs in this paper can be calculated according to the following formula:

(3) η=1−A/B

where η is the fusion ratio, *A* is the content of Cr or Ni in the weld metal, and *B* is the content of the Cr or Ni element in ER309L welding wire. When calculating, *A* and *B* take the composition content of the same element.

**Figure 11:**
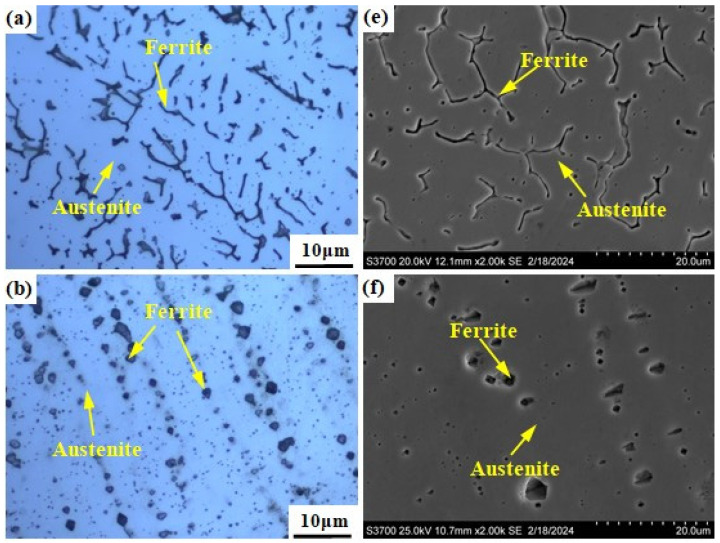

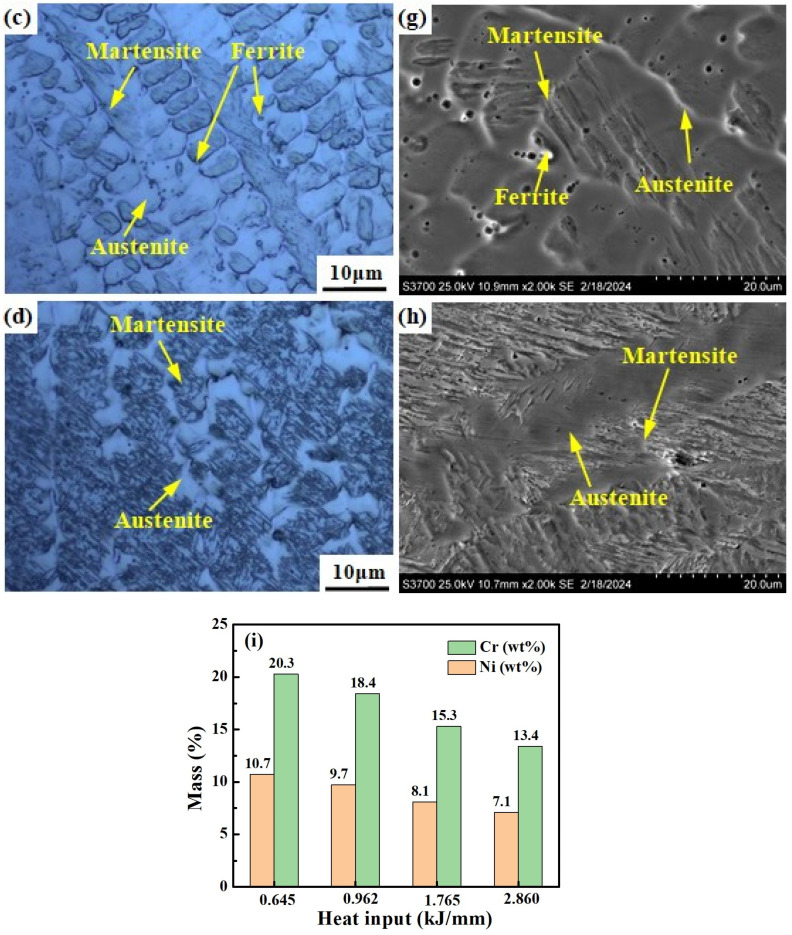
Microstructure of weld metals under different welding heat inputs: (**a**,**e**) 0.645 kJ/mm, (**b**,**f**) 0.962 kJ/mm, (**c**,**g**) 1.765 kJ/mm, (**d**,**h**) 2.860 kJ/mm. (**i**) Histograms of elements obtained from the positions shown in (**e**–**h**).

Figure 11 shows the microstructure of welded joints under different welding heat inputs observed by OM, SEM and SEM-EDS area scans. The microstructure of welding heat input with 0.645 kJ/mm and 0.962 kJ/mm consists e of the δ phase and A phase, with the δ phase distributed randomly in the A phase matrix (see Figure 11a,b,e,f). For the welding heat input of 0.645 kJ/mm and 0.962 kJ/mm, the morphology of the δ phase is shown to be dendritic-like, worm-like and globular-like, respectively. The microstructure of the welding heat input with 1.765 kJ/mm consists of the M phase, δ phase, and A phase. The δ phase is distributed in the A phase and the interface between the M phase and A phase (see Figure 11c,g). The microstructure of the welding heat input with 2.860 kJ/mm consists of the M phase and A phase (see Figure 11d,h). As the welding heat input increases, the content of the Cr and Ni elements in the weld metal gradually decreases (Figure 11i).

According to Equation (3), the fusion ratios for heat input 0.645 kJ/mm and 0.962 kJ/mm weld metals are 18% and 25%, respectively. The fusion ratios for 1.765 kJ/mm and 2.860 kJ/mm weld metals are 38% and 46%, respectively.

It is excellent that the fusion ratio of the weld metal with welding heat input of 0.645 kJ/mm and 2.860 kJ/mm is found to be basically consistent with the composition design of melted samples with 20% and 45% Q345B content, and the phase composition is also consistent. The slight difference in morphology of the δ phase may be due to the difference in cooling rate between the welding process and the smelting process. The composition design of the mixed-smelting samples can provide a reference for the preparation of welded joints, according to Table 3.

### 3.4. Corrosion Properties of Weld Metal

**Figure 12:**
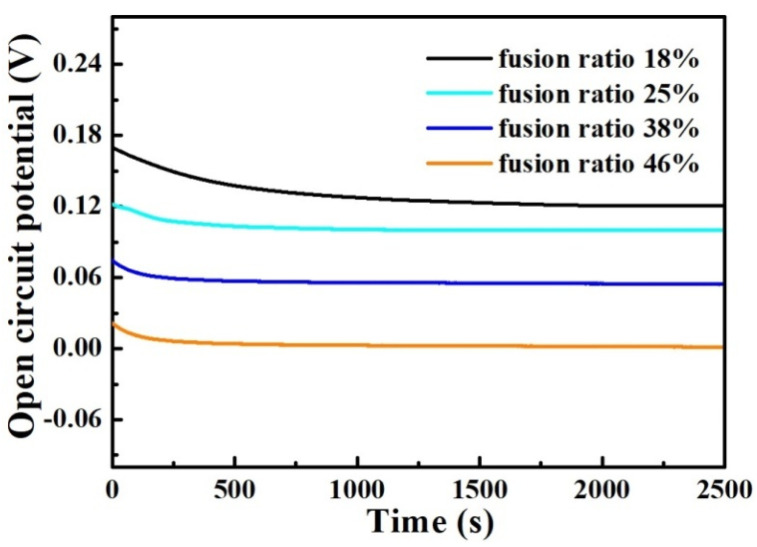
Variation in open-circuit potential over time for the weld-metal samples immersed in the 3.5 wt% NaCl solution.

Figure 12 shows the variation in open-circuit potential over time for the weld-metal samples immersed in the 3.5 wt% NaCl solution. As shown in Figure 12, the trend of the OCP-t curves for the different weld-metal samples is the same. During the immersion process, the OCPs of the weld-metal samples decrease at the beginning of immersion, and subsequently approach a constant value. As the fusion ratio increases, the OCP value gradually decreases, indicating that the corrosion resistance of the weld-metal samples gradually decreases. This law is consistent with the mixed-smelting samples.

**Figure 13:**
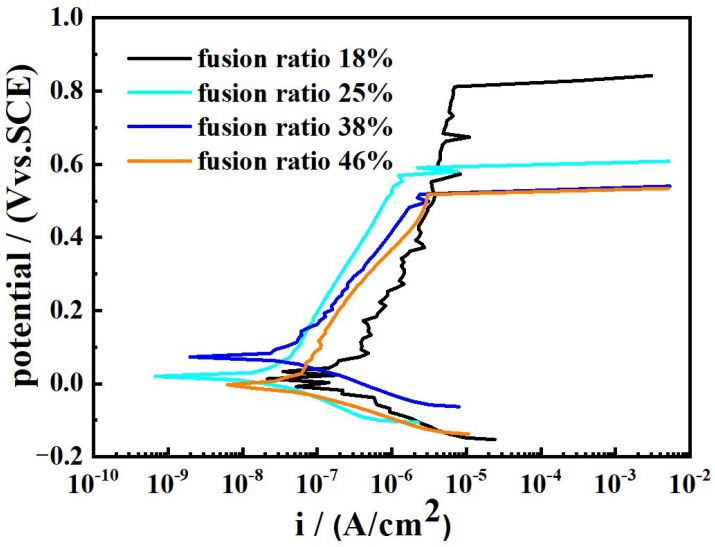
Potentiodynamic polarization plots of the weld-metal samples in the 3.5 wt% NaCl solution.

Figure 13 displays the potentiodynamic polarization plots of the weld-metal samples in the 3.5 wt% NaCl solution. All the samples exhibit similar features over the potential domain examined. The extracted data from Figure 13 are shown in Table 6. The distinct difference in the samples is the width of the passive region. The *E*_b_ − *E*_corr_ value for the samples with the fusion ratio of 18%, 25%, 38% and 46% is approximately 751 mV, 609 mV, 491 mV and 489 mV, respectively. The passivation region of the weld metal with the fusion ratio of 18% is significantly higher than that of the other samples. Moreover, as the fusion ratio increases, the *E*_corr_ value gradually decreases, while the *I*_corr_ value gradually increases. The much better corrosion resistance is obtained by the weld metal with 18% fusion ratio. It is mainly due to the fact that the fusion ratio of the base material and the welding wire is the lowest in the weld metal, and, accordingly, the content of the Cr and Ni elements is the highest. There is only the δ phase and A phase, no M phase in this weld metal, and the δ phase is distributed randomly in the A phase matrix.

**Figure 14:**
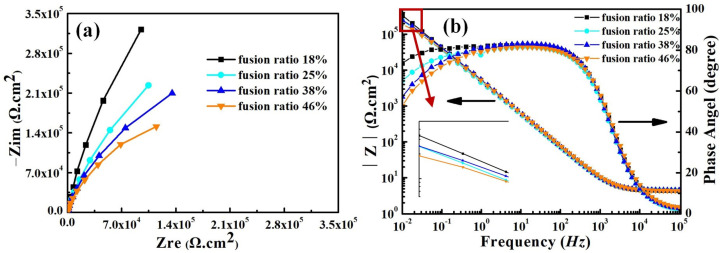
Electrochemical impedance spectra for weld-metal samples in the 3.5 wt% NaCl solution. (**a**) Nyquist plots, (**b**) Bode plots.

Figure 14 shows the electrochemical impedance spectra (EIS) of weld-metal samples in the 3.5 wt% NaCl solution. The Nyquist and Bode plots of all weld-metal samples have the same characteristics as the smelted samples (see Figure 9 and Figure 14). All weld-metal samples have a capacitance circuit and one time constant, and the fitted equivalent circuit is shown in Figure 10. The charge-transfer resistance and double-layer capacitance dominate the entire corrosion process for all weld-metal samples.

As the fusion ratio increases, the capacitance arc radius gradually decreases, which means that the corrosion resistance of the weld metal decreases gradually. This illustrates the fact that the increase in fusion ratio is detrimental to the corrosion resistance of the weld metal. There is good agreement between the measured (symbolic) experimental values and the fitted (solid-line) curve (see Figure 14a,b). Table 7 illustrates the EIS fitting parameters for the weld-metal samples. The range of χ^2^ values in Table 7 is 1.7 × 10^−5^ − 1.35 × 10^−4^, indicating that the fitted data have good fitting accuracy. According to Table 7, *R*_ct_ evidently decreases and *Q*_dl_ gradually increases with the increase in fusion ratio. As shown in Figure 14b, the maximum |z| of all weld-metal samples is obtained in the low-frequency range, and the maximum |z| gradually decreases with the increase in fusion ratio. This indicates that, as the fusion ratio increases, the corrosion resistance gradually decreases. This pattern is consistent with the results obtained from the mixed-smelting samples with different contents of Q345B.

**Figure 15:**
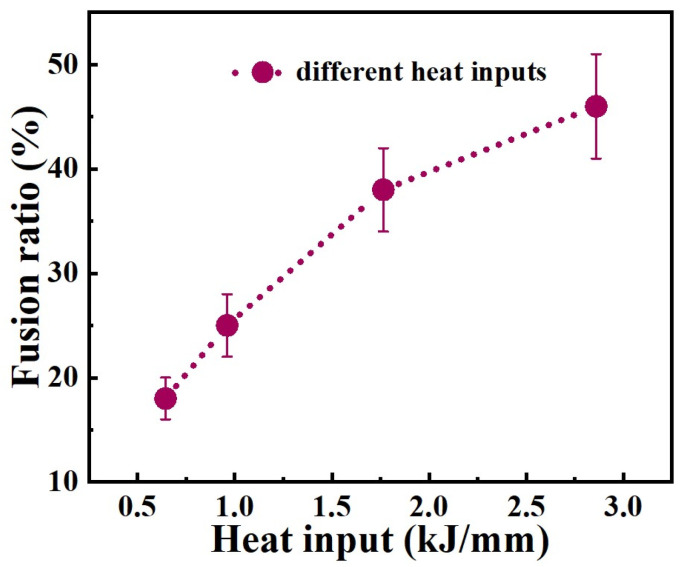
Fusion ratio as function of welding heat input.

As we know, the corrosion resistance of austenitic stainless steel is much higher than that of low-alloy steel. This is the main reason why the corrosion resistance of the weld metal decreases when the two materials were welded together. However, the welded joint obtained by connecting the two types of steel is widely used in manufacturing, to reduce costs. However, the microstructure and composition changes in dissimilar welded joints are very complex. In particular, the size of the weld metal formed by each welding process is relatively small, which poses great difficulty for the analysis of the microstructure. The research on the relationship between the organization structure and the corrosion performance of the welded joint is not deep enough. The composition and microstructure of weld metal is the main reason why the performance of welded joints is affected, and it is evidently influenced by the fusion ratio of the base materials and the welding wire. In this case, the new alloy composition was design by smelting Q345B and ER309L under different content proportions, in the present work. The composition of each new alloy reflects a weld component containing different fusion ratios. A relationship curve between fusion ratio and welding heat input was established, as shown in Figure 15. It is not hard to understand that the fusion ratio increases continuously with the welding heat input, because the high arc energy will facilitate the melting of the alloy. This indicates that the fusion ratio of the base material in weld metals could be adjusted by the welding heat input. The curve is also suitable for welding between other austenitic stainless steels and low-alloy steels. The composition of weld metal obtained with welding heat inputs of 0.645 kJ/mm and 2.860 kJ/mm is basically consistent with the designed alloy composition, and the microstructure and corrosion performance of the weld metal are basically consistent with the corresponding designed alloy composition. Based on this curve and the designed series of new alloy compositions, low-alloy steel and austenitic stainless steel dissimilar-welded joints that meet usage requirements can be prepared. For low-alloy steel and stainless steel dissimilar-welded joints used in tidal units, the base-metal fusion ratio of 20–30% can be selected to meet the extremely high corrosion-resistance requirements. The microstructure of the weld metal obtained from this variation interval consists of the ferrite phase and the austenite phase. The corrosion resistance of the weld metal of the welded joint is relatively high. The corresponding heat input in the curve ranges from 0.645 kJ/mm to 0.962 kJ/mm. It means that the heat input within this range can be selected for the welded-joint preparation. The selection of heat input for low-alloy steel and stainless steel dissimilar-welded joints applied to other environments can be carried out in the same way. This curve has significant guiding significance for engineering applications. However, due to the influence of multiple-layer multiple-passes, the microstructure in the weld metal may undergo changes. This may lead to a change in its corrosion-resistance performance.

From the above analysis, it can be found that the lower the Q345B content, the higher the corrosion resistance of the melted alloy. This is because the lower the Q345B content in the melted alloy is, the closer the composition of the melted alloy is to austenitic stainless steel. However, this does not mean that the low fusion ratio in the weld metal could cause the high corrosion resistance. When the heat input is adequately low during the welding, this will cause uneven wire laying and the formation of defects, and will result in reducing the corrosion resistance of the weld metal.

## 4. Conclusions

This study detailed the microstructure and electrochemical behavior of a designed alloy. By adjusting the welding-process parameters, the composition of the weld metal could be obtained by matching with the designed mixed alloy, and the microstructure and electrochemical properties of the weld metal were also been studied. Based on the present investigation, the main conclusions that can be drawn are the following:

(1) The composition of the new alloy is obtained by mixed-smelting Q345B low-alloy steel with the different contents and with ER309L stainless steel. The contents of Q345B have the distinct influence on the composition, morphology and corrosion performance of the melted alloys.
(2) The microstructure of the melted alloys containing 20% to 30% Q345B consists of the ferrite (δ) phase and austenite (A) phase, the samples containing 45% to 50% Q345B consists of the martensite (M) phase and austenite (A) phase, and the samples containing 40% Q345B consist of the martensite (M) phase, ferrite phase (δ-phase), and austenite (A) phase.
(3) The fusion ratio between Q345B and ER309L can be changed by the welding heat input during the GMAW process. When the welding heat input is 0.645 kJ/mm and 2.860 kJ/mm, respectively, the composition of the weld metal is consistent with the melted alloy containing 20% and 45% Q345B, and the microstructure and corrosion resistance are also basically equivalent.
(4) All mixed-smelting samples and weld-metal samples exhibit one capacitance loop in their Nyquist plots, and one time constant can be observed during the experimental process. Potentiodynamic polarization and EIS results confirm that the corrosion resistance of samples decreased gradually with the increase in the mixed-smelting ratio for the melted alloy and the welding heat input for the weld metal.
(5) A relationship curve between the fusion ratio and welding heat input is established. The composition of the weld metal obtained with welding heat inputs of 0.645 kJ/mm and 2.860 kJ/mm is basically consistent with the designed alloy composition. Based on this curve and the designed series of new alloy compositions, low-alloy steel and austenitic stainless steel dissimilar-welded joints that meet usage requirements can be prepared. This curve has important guiding significance for engineering applications.

## Figures and Tables

**Table 1:** Chemical composition of Q345B and ER309L (wt%).

Metal	C	Si	Mn	Cr	Ni	Mo	Cu	Fe
Q345B	0.1	0.29	1.24	0.077	0.032	0.0061	0.095	Bal.
ER309L	0.032	0.48	2.11	24.7	13	-	0.004	Bal.

**Table 2:** The welding parameters of ER309L welding wire deposited on Q345B.

Test	Voltage (V)	Current (A)	Speed (mm/s)	Heat Input (kJ/mm)
1	19	140	3.3 ± 0.2	0.645
2	21	189	3.3 ± 0.2	0.962
3	28	260	3.3 ± 0.2	1.765
4	34	347	3.3 ± 0.2	2.860

**Table 3:** Chemical composition of the prepared samples under different smelting ratios (wt%).

Smelting Ratios	C	Si	Mn	Cr	Ni	Mo	Cu	Fe	Creq	Nieq
20%	0.044	0.45	1.95	20.27	10.67	0.0011	0.021	Bal.	20.27	12.97
30%	0.052	0.42	1.85	17.31	9.11	0.0018	0.031	Bal.	17.32	11.61
40%	0.059	0.40	1.76	14.85	7.81	0.0024	0.04	Bal.	14.86	10.47
45%	0.063	0.39	1.72	13.62	7.16	0.0027	0.04	Bal.	13.63	9.90
50%	0.066	0.39	1.68	12.39	6.52	0.0031	0.05	Bal.	12.40	9.33

**Table 4:** The electrochemical data obtained from Figure 7.

Smelting Ratio (%)	*β_a_ (mV/Decade)	**β_c_ (mV/Decade)	*I*_corr_ (10^−8^ A/cm^2^)	*E*_corr_ (mV vs. SCE)	*E*_b_ (mV vs. SCE)	*E*_b_ − *E*_corr_ (mV)
20	130	−99.2	7	101	583	482
30	174	−92.4	11	89	520	431
40	219	−83.4	12	69	489	420
45	245	−82.4	15	59	335	276
50	250	−81.7	16	58	325	267

*β_a_ is the Tafel slope of the anodic reaction. **β_c_ is the Tafel slope of the cathodic reaction.

**Table 5:** EIS fitting parameters for the prepared samples under different Q345B contents.

Smelting Ratio (%)	*R*_s_ (Ω cm^2^)	*R*_ct_ (kΩ cm^2^)	*Q*_dl_ (10^−5^ s^n^ Ω^−1^ cm^−2^)	*n*_dl_	χ^2^ 10^−4^
20	8	558	3.00	0.931	1.04
30	5	412	3.91	0.911	1.93
40	5	261	4.35	0.934	1.27
45	9	258	4.92	0.938	0.86
50	9	165	5.86	0.931	4.27

**Table 6:** The electrochemical data obtained from Figure 13.

Fusion Ratio (%)	*β*_a_ (mV/Decade)	*β*_c_ (mV/Decade)	*I*_corr_ (10^−8^ A/cm^2^)	*E*_corr_ (mV vs. SCE)	*E*_b_ (mV vs. SCE)	*E*_b_ − *E*_corr_ (mV)
18	258	−60.5	4	63	814	751
25	262	−67.6	6	51	660	609
38	314	−72.5	10	30	521	491
46	354	−78.6	13	28	517	489

**Table 7:** EIS fitting parameters for weld-metal samples.

Fusion Ratio (%)	*R*_s_ (Ω cm^2^)	*R*_ct_ (kΩ cm^2^)	*Q*_dl_ (10^−5^ s^n^ Ω^−1^ cm^−2^)	*n*_dl_	χ^2^ 10^−4^
18	4	859	3.51	0.9213	0.17
25	4	590	3.72	0.9071	0.8
38	5	405	3.84	0.9228	1.35
46	4	315	4.14	0.9032	1.34

## Data Availability

The original contributions presented in the study are included in the article, further inquiries can be directed to the corresponding authors.
